# Curcumin inhibits proliferation of gastric cancer cells by impairing ATP-sensitive potassium channel opening

**DOI:** 10.1186/1477-7819-12-389

**Published:** 2014-12-19

**Authors:** Xiaohong Liu, Kai Sun, Ailin Song, Xiaoyun Zhang, Xu Zhang, Xiaodong He

**Affiliations:** Department of General Surgery, second affiliated hospital of Lanzhou University, Lanzhou, Gansu 730000 China; The Second Department of Thoracic Surgery, First Affiliated Hospital of Xi’an Jiaotong University, Xi’an, Shaanxi 710061 China; Department of Pathology, Lanzhou University Medical School, 199 West Donggang Road, Lanzhou, Gansu 730000 China; Lanzhou University, 199 West Donggang Road, Lanzhou, Gansu 730000 China

**Keywords:** apoptosis, curcumin, gastric cancer, K_ATP_

## Abstract

**Background:**

This study was aimed to investigate whether ATP-sensitive potassium channel (K_ATP_) is involved in curcumin’s anti-proliferative effects against gastric cancer.

**Methods:**

In an *in vitro* study, gastric cancer cell line SGC-7901 was treated with curcumin at serial concentrations and co-administrated with the K_ATP_ opener, diazoxide. The effect of curcumin and diazoxide on proliferation were assessed by MTT assay. Mitochondrial membrane potential (MMP) was studied by flow cytometry detection of rhodamine 123 staining. Apoptosis was evaluated by flow cytometry detection of Annexin V propidium iodide double staining. In an *in vivo* study, SGC-7901 cells were planted into nude mice as xenografts. Animals were treated with curcumin co-administered with diazoxide. Tumor volume and tumor weight were observed.

**Results:**

Curcumin incubation significantly induced loss of MMP in SGC-7901 cells in a dose- dependent manner (*P* < 0.05); the cell apoptotic rate also dramatically increased after curcumin incubation in a dose-dependent manner (*P* < 0.05). After co-administration with diazoxide, however, we found that both the MMP-loss-inducing and the apoptosis-inducing effects of curcumin in SGC-7901 cells were significantly impaired (all *P* < 0.05). As a result, the proliferation of SGC-7901 cells was maintained by diazoxide treatment.

**Conclusions:**

Impaired mitoK_ATP_ opening causes MMP loss, and is involved in curcumin-induced apoptosis in gastric cancer.

## Background

As the third leading cause of death in men and fourth in women with malignant tumors, gastric cancer is now threatening people’s lives worldwide [1]. Gastric cancer is one of the highly malignant carcinomas arising from epithelial cells, and is characterized by therapeutic inefficiency and poor prognosis [2]. Like most malignant tumors, the malignancy of gastric cancer is caused by uncontrolled cell proliferation, invasion, and metastasis. The therapeutic options for gastric cancer are often limited because most patients with gastric cancer are diagnosed at an advanced stage [3]. Currently, chemotherapy and radiotherapy are employed to treat gastric cancer at an advanced stage. However, the side effects of these therapies restrict their application and lower the quality of life of patients [4]. Traditional Chinese medicine has been effectively applied in treating malignant diseases for a long time in Eastern Asia [5]. Several monomers, including curcumin, discovered from traditional Chinese medicine formulas have been shown to have anti-cancer activities in previous studies [6]. However, the molecular mechanisms for these activities are still unclear.

Natural extracts or products have attracted attention and become the focus of anti-cancer drug research because over two-thirds of novel anti-cancer drugs discovered in recent decades are of natural original [7]. Curcumin, also known as diferuloylmethane, is extracted from the rhizome of *Curcuma longa*. The spectrum of biological activities of curcumin is thought to be wide, including anti-angiogenic, anti-oxidant, anti-inflammatory and anti-diabetic effects [8]. Previous studies have shown that the expression of oncogenes [9], transcription factors [10], cytokines, and growth factors [11] was modulated in the anti-cancer effects of curcumin. Although several mechanisms regulating proliferation and apoptosis of curcumin have been investigated, many more studies are still needed.

ATP-sensitive potassium channels, also referred to as K_ATP_, are distributed throughout the organs and tissues, including skeletal muscle, cardiac muscle, brain, and kidney [12]. K_ATP_ is characterized by a hetero-octameric molecular structure; it is composed of four Kir6 and four sulphonylurea receptor subunits [13]. It is now believed that the fundamental function of K_ATP_ is to adjust membrane excitability to cellular metabolic status [14]. K_ATP_ located on the mitochondrial membrane is called mitoK_ATP_, the opening of which reduces the mitochondrial transition opening permeability by increasing the activity of inwardly rectifying potassium channels under stressful conditions [15, 16]. Thus, opening of the mitoK_ATP_ could attenuate cell apoptosis by maintaining mitochondrial membrane potential (MMP) [17].

A recent study preliminarily established the relationship between mitoK_ATP_ and the proliferation of malignant cancer cells, such as glioma cells [18]. This association aroused our interests in exploring whether the possibility that curcumin’s anti-cancer effect on gastric cancer is related to mitoK_ATP_. In this study, we investigated the effect of curcumin and the selective mitoKATP opener diazoxide on proliferation, mitochondrial transition opening permeability, and apoptosis in human gastric cancer cells SGC-7901 *in vitro*. A corresponding *in vivo* study was also implemented. Our results will contribute to a deepened understanding of the molecular mechanisms of curcumin’s anti-cancer activity.

## Methods

### Cell culture and treatment

Human gastric cancer cell line SGC-7901 was purchased from the American Type Culture Collection and cultured in DMEM (Gibco) supplemented with 10% FBS (Gibco). The cells were maintained in a humidified cell incubator (Thermo Scientific, Pittsburgh, PA, USA) containing 5% CO_2_ at 37°C. Equal numbers of cells were divided into seven independent groups: a control group (C), a low-dose curcumin group (LCur), a medium-dose curcumin group (MCur), a high-dose curcumin group (HCur), a low-dose curcumin group treated with diazoxide (LCur + DZ), a medium-dose curcumin group treated with diazoxide (MCur + DZ) and a high-dose curcumin group treated with diazoxide (HCur + DZ).

In the control group, cells were maintained in culture medium, as described; in LCur, cells were treated with curcumin (Sigma-Aldrich, St. Louis, MO, USA) solution at concentration of 15 μmol/l; in MCur, cells were treated with curcumin solution at a concentration of 30 μmol/l; in HCur, cells were treated with curcumin at a concentration of 60 μmol/l; in LCur + DZ, cells were treated with diazoxide (Sigma-Aldrich) at a concentration of 100 μmol/l together with curcumin at a concentration of 15 μmol/l; in MCur + DZ, cells were treated with diazoxide at a concentration of 100 μmol/l together with curcumin at a concentration of 30 μmol/l; in HCur + DZ, cells were treated with diazoxide at concentration of 100 μmol/l together with curcumin at a concentration of 60 μmol/l.

### Cell proliferation assessment

A 3-(4,5-dimethylthiazol-2-yl)-2-5-diphenyltetrazolium-bromide (MTT) assay was employed to assess the proliferation of SGC-7901 cells. Briefly, 1 × 10^4^ cells per well were planted in a 96-well culturing plate (Corning Costar, Corning, NY, USA) for 24 hours and then treated with diazoxide and curcumin, as described. Then 20 μl MTT (Sigma-Aldrich, 5 mg/ml, dissolved in PBS) was added to each well and 150 μl dimethylsulfoxide (Sigma-Aldrich) was added to replace medium from each well. Absorbance at 450 nm (*A*_450_) was measured using a plate reader (Bio-Rad, Hercules, CA, USA). The growth inhibition rate was calculated using the formula:


### Cell apoptosis assay

The apoptosis assessment of SGC-7901 cells in each group was carried out by flow cytometry using an Annexin V-FITC Apoptosis Detection Kit (BD, San Jose, CA, USA) as described previously [19]. Briefly, equal numbers of cells from each group were washed by PBS and titrated by binding buffer to concentrations of 1 × 10^6^ cell/ml. Then 100 μl of this cell suspension, 5 μl Annexin V-FITC solution and 5 μl propidium iodide were added to a culture tube for 15 min incubation in a dark chamber. After 400 μl binding buffer was added to each tube, apoptosis was then analyzed by a fluorescence-activated cell sorting flow cytometer (BD, San Jose, CA, USA).

### Mitochondrial membrane potential (MMP) detection

The MMP in SGC-7901 cells was determined by detection of rhodamine 123 staining by flow cytometry following the protocols described in a previous study [19]. Cells from each group were washed by PBS twice then titrated to 1 × 10^6^ cells/ml. Rhodamine 123 solution (Beyotime, Shanghai, China) was then added to the cell suspension at a final concentration of 1 μmol/l. After incubation at 37°C in a dark chamber for 30 min, the fluorescent signal of rhodamine 123 released from cells was analyzed by a fluorescence-activated cell sorting flow cytometer (BD, San Jose, CA, USA) at 529 nm.

### *In vivo*xenograft tumor study

Cultured SGC-7901 cells were suspended and titrated in PBS at a concentration of 1 × 10^7^/ml. 100 μl of the cell suspension was subcutaneously injected into the right armpits of male BALB/C nude mice (Animal Experimental Center of the Fourth Military Medical University) with a mean body weight of (17.64 ± 5.19) g. After the volumes of the xenograft tumors exceeded 100 mm^3^, 18 mice were divided into three independent groups randomly and evenly (six mice per group); namely, a control group (C), a curcumin treatment group (Cur) and curcumin and diazoxide co-treatment group (Cur + DZ). In Cur + DZ, mice received peritumoral injections of curcumin (50 mg/kg) applied together with peritumoral injections of diazoxide (10 mg/kg) twice per day for 2 weeks. In Cur, mice received peritumoral injections of curcumin (50 mg/kg) twice per day for 2 weeks. In C, mice received peritumoral injections of physiological saline twice per day for 2 weeks. The animal treatment protocols were in accordance with our pre-experimental results (not shown) and several previous studies [20, 21]. The volume of tumor was measured twice per week and calculated by the formula:


The tumor was also weighted after the mice were sacrificed. All animal experimental procedures were performed in accordance with the National Institutes of Health Guide for the Care and Use of Laboratory Animals.

### Statistical considerations

All data collected in this study are expressed as mean ± standard deviation. The statistical analysis was processed by software SPSS (version 16.0) by analysis of variance (ANOVA), least-significant difference and Fisher exact tests. *P* < 0.05 was considered statistically significant when comparing differences.

## Results

### Curcumin inhibited proliferation of SGC-7901 cells but this was alleviated by diazoxide co-administration

**Figure 1:**
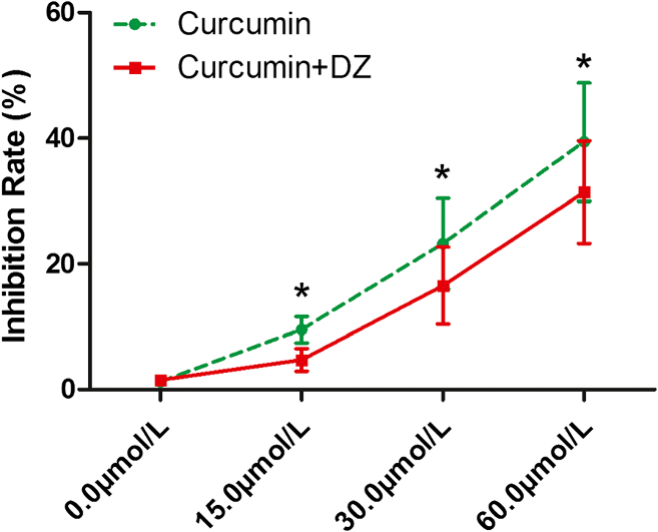
**Effects of curcumin and its co**-**administration with diazoxide on proliferation of SGC**-**7901 cells.** Cell proliferation was assessed by MTT assay. Graphs shows inhibition rate of SGC-7901 cells treated by curcumin (red) and co-administration of curcumin and diazoxide (green). Values are represented as mean ± standard deviation. *, values are significantly different when compared with control; #, values are significantly different when compared with curcumin treatment.

As shown in Figure 1, according to the results of the MTT assay, curcumin elevated inhibition rate in a concentration-dependent manner in LCur (9.5% ± 2.10%), MCur (23.2% ± 7.21%), and HCur (39.4% ± 9.43%) compared with C (1.20% ± 0.25) (all *P* < 0.05). However, after co-administration of diazoxide, the inhibition rate was decreased in LCur + DZ (6.72% ± 1.80%), MCur + DZ (16.55% ± 6.11%), and HCur + DZ (31.43 ± 8.15%) compared with corresponding LCur, MCur, and HCur (all *P* < 0.05).

### Co-administration of diazoxide impaired curcumin’s effects of decreasing mitochondrial membrane potential (MMP) in SGC-7901 cells

**Figure 2:**
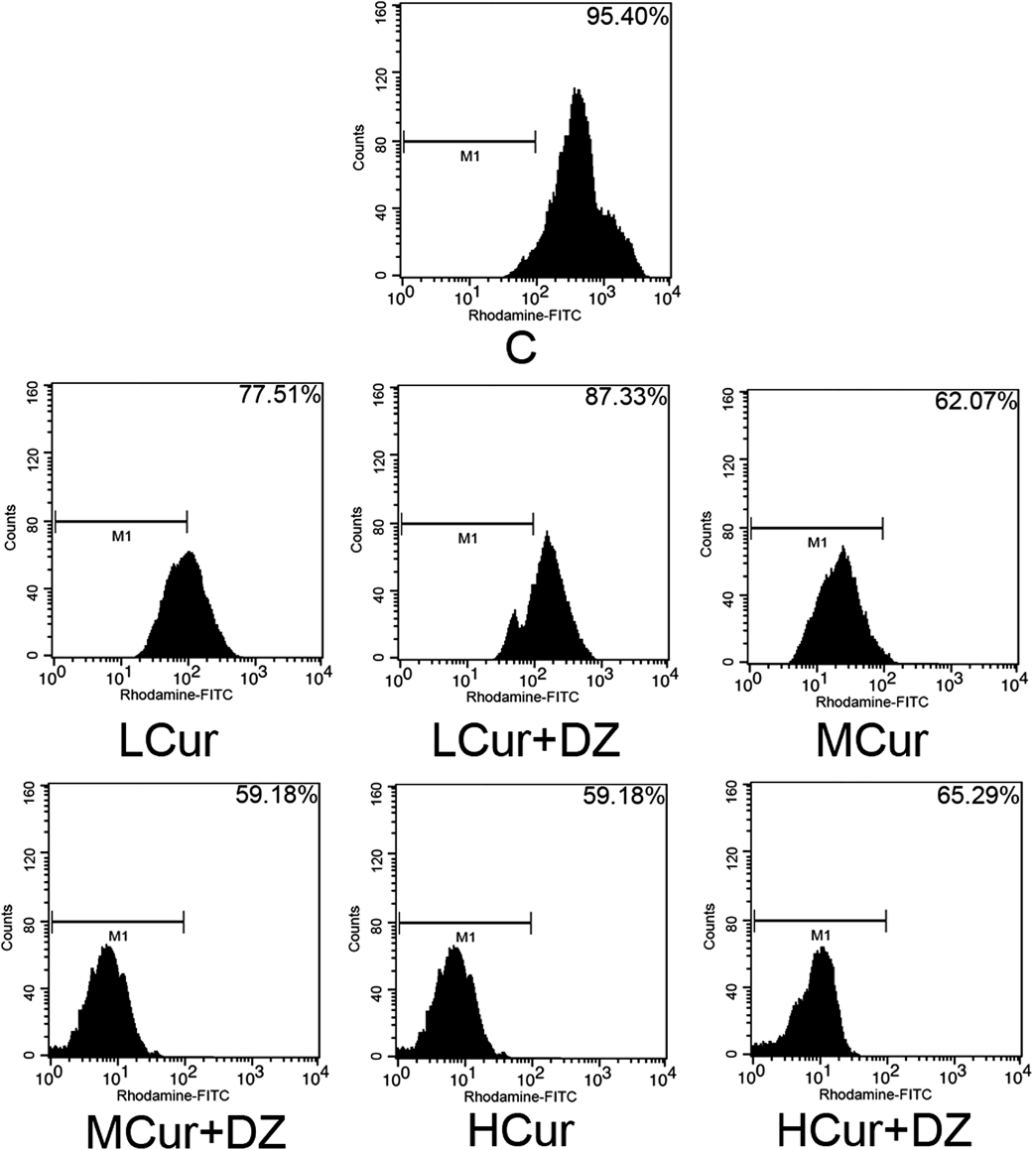
**Effects of curcumin and its co**-**administration with diazoxide on mitochondrial membrane potential in cultured SGC**-**7901 cells.** Cells were stained with rhodamine 123 and then detected by a flow cytometer. Cells with decreased MMP show less detected fluorescence from rhodamine 123. Charts of flow cytometry from top to bottom and from left to right show the detected rhodamine 123 fluorescence of C, LCur, LCur + DZ, MCur, MCur + DZ, HCur, and HCur + DZ. C, control; DZ, diazoxide; FITC, fluorescein isothiocyanate; HCur, high-dose curcumin; LCur, low-dose curcumin; MCur, medium-does curcumin; MMP, mitochondrial membrane potential.

In a concentration-dependent manner (Figure 2), curcumin lowered MMP in SGC-7901 cells in LCur (77.51% ± 8.13%), MCur (62.07% ± 4.52%), and HCur (59.18% ± 9.30%) compared with C (95.40% ± 7.62%) (all *P* < 0.05). However, MMP was significantly higher with the co-administration of diazoxide; LCur + DZ (87.33% ± 4.68%), MCur + DZ (59.18% ± 5.92%), and HCur + DZ (65.29% ± 6.10%) (all *P* < 0.05).

### Co-administration of diazoxide mitigated apoptosis in curcumin-incubated SGC-7901 cells

**Figure 3:**
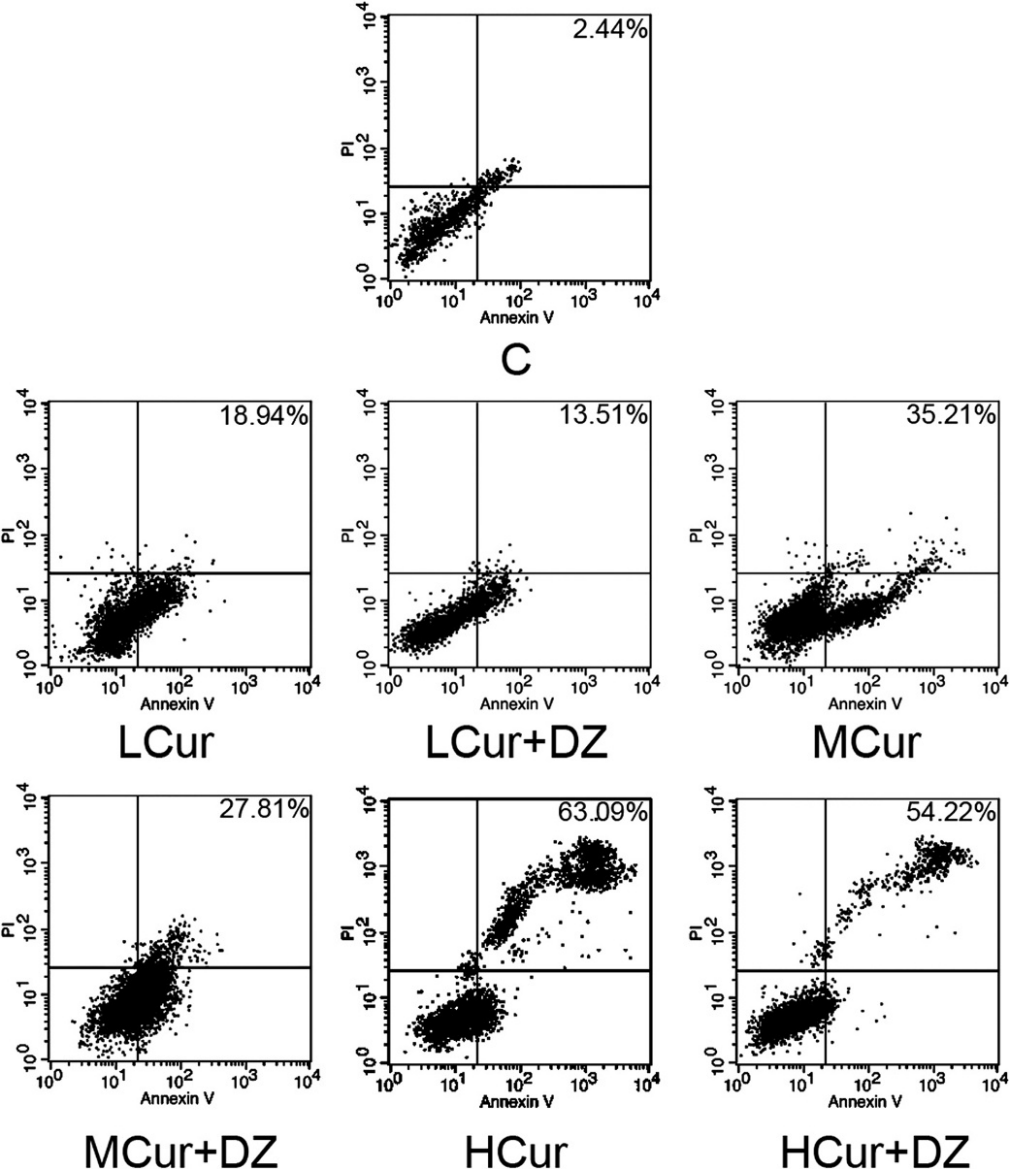
**Effects of curcumin and its co**-**administration with diazoxide on apoptosis of SGC**-**7901 cells.** Cells were double-stained with Annexin V and propidium iodide, and apoptosis rates were analyzed by flow cytometry. Percentage on the right top corner of each chart stands for apoptosis rate. Charts of flow cytometry from top to bottom, from left to right show the detected rhodamine 123 fluorescence of C, LCur, LCur + DZ, MCur, MCur + DZ, HCur and HCur + DZ, respectively. C, control; DZ, diazoxide; HCur, high-dose curcumin; LCur, low-dose curcumin; MCur, medium-does curcumin, PI; propidium iodide.

Detected by flow cytometry, compared with C (2.44% ± 0.79%) the apoptosis rate of SGC-7901 cells was increased by curcumin incubation in a concentration-dependent manner in LCur (18.94% ± 2.32%), MCur (35.21% ± 2.77%), and HCur (63.09% ± 3.05%) (all *P* < 0.05). When applied together, diazoxide alleviated the curcumin-induced apoptosis in LCur + DZ (13.51% ± 1.85%), MCur + DZ (27.81% ± 2.24%), and HCur + DZ (54.22% ± 2.72%). The results are shown in Figure 3.

### Growth of xenograft tumor was reduced by curcumin but reversed by co-administration of diazoxide *in vivo*

**Figure 4:**
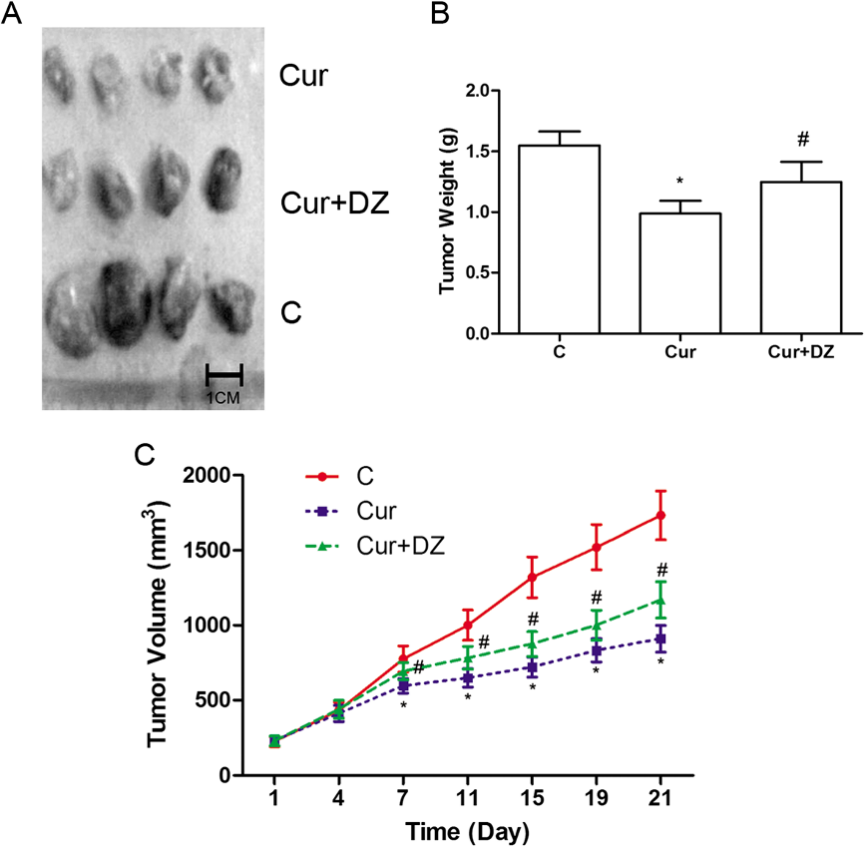
**Effects of curcumin and its co**-**administration with** **diazoxide** **on growth of SGC**-**7901 tumor xenografts. (A)** Isolated tumor xenografts from nude mice of Cur, Cur + DZ, and C at day 21. **(B)** Weight of isolated xenografts from nude mice of Cur, Cur + DZ, and C at day 21. **(C)** Tumor volume of SGC-7901 tumor xenografts in Cur (blue), Cur + DZ (green) and C (red) from day 1 to day 21. Values are represented as mean ± standard deviation. *, values are significantly different compared with C; #, values are significantly different compared with Cur. C, control; Cur, curcumin; DZ, diazoxide.

At the end of 3 weeks after initiation of curcumin and diazoxide treatment, all mice were alive. After sacrifice, tumors were isolated. Compared with C, tumor volume was significantly reduced in Cur starting from day 7 (*P* < 0.05). However, tumor volume in Cur + DZ was significantly higher than that in Cur (*P* < 0.05). In Cur, curcumin administration showed a significant inhibitory effect on the weight of xenografts compared with C (*P* < 0.05). However, this effect was mitigated by co-administration of diazoxide (*P* < 0.05). The results are shown in Figure 4.

## Discussion

We explored the involvement of K_ATP_ in the anti-proliferation effects of curcumin in gastric cancer. It was found that curcumin could inhibit the proliferation of gastric cancer cells by inducing apoptosis. Our *in vitro* results showed that curcumin-induced apoptosis of SGC-7901 cells by facilitating the collapse of MMP, which was believed to initiate the mitochondria-dependent apoptotic pathway. However, the co-administration of diazoxide, which is a mitoK_ATP_ selective opener, alleviated the collapse of MMP in curcumin-incubated SGC-7901 cells. In our *in vivo* study, the reduction in both volume and weight of xenograft tumor by curcumin was also reversed by co-administration of diazoxide. These results indicated that curcumin could induce apoptosis of gastric cancer cells via deactivating mitoK_ATP_, which would expedite the collapse of MMP.

With the improvement of modern medical technology and cancer prevention, the incidence of gastric cancer has decreased remarkably in the past few years [22]. However, globally, gastric cancer is now the second leading cause of mortality in malignant diseases [1]. The prognosis of patients with gastric cancer is poor, especially in patients with metastatic lymph nodes and low serum albumin levels, who are considered not suitable for surgical treatment [23]. Owing to the unapparent and sneaky clinical manifestations of early stage gastric cancer, patients are often only diagnosed when the cancer is at an advanced stage [24]. Thus, the current most curative therapy, surgery [25], is excluded from treatment strategies. Alternative therapies, including chemotherapy, radiotherapy, and radiochemotherapy, though effective, are none of them curative. In recent decades, several natural products originating from medicinal herbs have broadened our understanding because of their extensive biological activities [26]. Such drugs as emodin [27], curcumin [28], and matrine [29] have been demonstrated to have anti-cancer effects by inhibiting proliferation, invasion, and metastasis of multiple malignant cancers. Although many studies revealed their pharmacological mechanisms, much research is still needed.

Used as a coloring agent, spice, and flavoring, curcumin has also been widely applied since ancient times in medical systems in Eastern and Southeastern Asia as an important ingredient of medicinal formulas [30]. Modern medical studies found that this bioactive agent, extracted from the rhizome of a herb named *Curcuma Longa Linn*, exerted potent anti-cancer activity by inhibiting cell proliferation *in vitro* and *in vivo*[31]. The mechanisms involved in curcumin’s proliferation inhibition were thought to be complicated: multiple pathways and molecular mediators were proved relevant. It was believed that three canonical apoptotic pathways, namely the death receptor [32], mitochondrial [33], and endoplasmic reticulum stress [34] pathways, were activated to induce apoptosis of cancer cells. Some transcriptional factor-related mechanisms, such as downregulation of TNF-induced nuclear factor κB mediated gene expressions [35] by curcumin were also indicated [36]. A recent report of the involvement of mitoK_ATP_ in regulating proliferation, invasion, and metastasis in glioma cells [37] provided new clues to the mechanisms of curcumin’s anti-proliferation effects.

It was suggested that K_ATP_ channels were distributed throughout the body, located on cell membranes and mitochondrial membranes, including malignant cells [38]. The major function of mitoK_ATP_ was supposed to be to adjust mitochondrial membrane functions to external stressors by opening the ion channel. Most studies concerning mitoK_ATP_ have been on cardiac ischemia-reperfusion injuries [39]. Results from these studies suggest that opening of mitoK_ATP_ channels could protect against apoptosis [40]. At the early stage of apoptosis, the opening of mitoK_ATP_ could inhibit depolarization of mitochondrial membrane to maintain MMP [41]. Thus, the mitochondrial membrane was stabilized to prevent further apoptotic chain reaction, such as transition pore formation, cytochrome *c* release or caspase cascade activation. Diazoxide is a widely used antihypertensive agent that acts as a selective opener of mitoK_ATP_ to modulate the loss of MMP [42, 43]. As a result, diazoxide could further protect cells from apoptosis.

In this study, to test the participation of mitoK_ATP_ in curcumin-induced apoptosis of SGC-7901 cells, diazoxide was co-administrated with curcumin to incubate SGC-7901 cells. The results of an *in vitro* study indicated that diazoxide co-administration alleviated the apoptosis of SGC-7901 by stabilizing MMP. The co-administration of diazoxide impaired curcumin’s inhibitory effects against xenograft tumor growth. The results of an *in vivo* study consolidated the findings of the *in vitro* study that impaired mitoK_ATP_ is one of the possible mechanisms of curcumin’s anti-proliferative effects against gastric cancer.

## Conclusions

We can conclude that:

1. Curcumin inhibits proliferation of gastric cancer by inducing apoptosis.
2. Impaired mitoK_ATP_ opening causes MMP loss, and is involved in curcumin-induced apoptosis in gastric cancer.

## Abbreviations

C: control
Cur: curcumin
DMEM: Dulbecco’s modified Eagle’s medium
DZ: diazoxide
FBS: fetal bovine serum
HCur: high-dose curcumin
K_ATP_: ATP-sensitive potassium channel
LCur: low-dose curcumin
MCur: medium-does curcumin
MMP: mitochondrial membrane potential
MTT: 3-(4,5-dimethylthiazol-2-yl)-2-5-diphenyltetrazolium-bromide
PBS: phosphate-buffered saline
TNF: tumor necrosis factor.
